# Treg cell-derived exosomes miR-709 attenuates microglia pyroptosis and promotes motor function recovery after spinal cord injury

**DOI:** 10.1186/s12951-022-01724-y

**Published:** 2022-12-13

**Authors:** Wu Xiong, Cong Li, Guang Kong, Qiang Zeng, Siming Wang, Guoyong Yin, Jun Gu, Jin Fan

**Affiliations:** 1 The First Affiliated Hospital of Nanjing Medical University, 300 Guangzhou Road, Nanjing, Jiangsu China; 2 Nanjing Medical University, 101 Longmian Road, Nanjing, Jiangsu China; 3 Department of Orthopedics, Xishan People’s Hospital, Wuxi, 214000 China

**Keywords:** Spinal cord injury, Treg cell, Pyroptosis, Exosomes, Neuroinflammation

## Abstract

Neuroinflammation is an important cause of poor prognosis in patients with spinal cord injury. pyroptosis is a new type of inflammatory cell death. Treg cells has been shown to play an anti-inflammatory role in a variety of inflammatory diseases, including inflammatory bowel disease, amyotrophic lateral sclerosis, and arthritis. However, little is known about Treg cells' potential role in pyroptosis following spinal cord injury. The aim of this research was to look into the effect of Treg cells to motor function recovery, pyroptosis and the mechanism behind it after SCI. Here, we found that pyroptosis mainly occurred in microglia on the seventh day after spinal cord injury. Konckout Treg cells resulted in widely pyroptosis and poor motor recovery after SCI. In conversely, over-infiltration of Treg cell in mice by tail vein injection had beneficial effects following SCI.Treg cell-derived exosomes promote functional recovery by inhibiting microglia pyroptosis in vivo. Bioinformatic analysis revealed that miRNA-709 was significantly enriched in Treg cells and Treg cell-secreted exosomes. NKAP has been identified as a miRNA-709 target gene. Moreover, experiments confirmed that Treg cells targeted the NKAP via exosomal miR-709 to reduce microglia pyroptosis and promote motor function recovery after SCI. More importantly, The miR-709 overexpressed exosomes we constructed significantly reduced the inflammatory response and improved motor recovery after spinal cord injury. In brief, our findings indicate a possible mechanism for communication between Treg cells and microglia, which opens up a new perspective for alleviating neuroinflammation after SCI.

**Supplementary Information:**

The online version contains supplementary material available at 10.1186/s12951-022-01724-y.

## Introduction

SCI is a devastating injury that frequently results in severe limb dysfunction below the injured segment or even death [1]. Complicated systems of crosstalk between the CNS and the immune system have developed to alleviate the effect of SCI and faciliate recovery [2]. After spinal cord injury, both innate and adaptive immune cells are activated and perform critical roles in debris removal and inflammation resolution [3]. However, neuroinflammation resulting from immoderate or uncontrollable immune activation can cause secondary damage and impede the repair process of spinal cord injury [4]. As a result, creating an immune environment conducive to tissue repair is critical for neurological recovery following spinal cord injury.

Microglia are resident tissue macrophages in the central nervous system [5]. During development, microglia remove apoptotic cells and abnormal neural connections, thereby maintaining homeostasis of the spinal cord microenvironment [6, 7]. After spinal cord injury microglia rapidly accumulate to the site of injury and activated microglia cause neuroinflammatory secondary injury by releasing proinflammatory mediators such as reactive oxygen species and proinflammatory cytokines [8, 9]. Previous research has indicated that inhibiting the dysregulated neuroinflammatory cascade response following SCI contributes to the improvement of motor function in patients with spinal cord injury [10, 11]. Neuroinflammation after CNS injury is thought to be mediated by activated inflammasomes [12, 13]. The inflammasome is composed of pattern recognition receptors (PRRs), pathogen-associated molecular patterns (PAMPs), and damage-associated molecular patterns (DAMPs), which promotes pro-inflammatory caspase activation and gasdermin D (GSDMD) cleavage, resulting in pyroptosis [14, 15].

Pyroptosis is a type of pro-inflammatory programmed cell death that is essentially an uncontrollable inflammatory damage caused by the organism's excessive response to external stimuli, and is closely related to the oxidative stress level, immune response, and other intracellular environmental homeostasis [16, 17]. Danger signals are transmitted intracellularly following injury to activate inflammasomes such as NLRP3 [18]. The activated inflammasome then cleaves and activates caspase-1, cleaving and isolating the GSDMD. The GSDMD N-terminal fragment is transferred to the plasma membrane and forms a membrane pore, causing cell swelling, rupture, and the release of inflammatory mediators [19, 20]. The released inflammatory mediators attract more immune cells, resulting in an inflammatory cascade [21]. As a result, preventing pyroptosis after a spinal cord injury can help alleviate secondary inflammatory injury and thus improve patient prognosis [22]. Although pyroptosis of microglia has been mentioned in a number of neuroinflammation-related diseases, the mechanism of its occurrence, particularly in spinal cord injury, is unknown.

Treg cells are a small subset of CD4 + T cells distinguished by the expression of a number of signature proteins, including CD25, Foxp3, and Helios [23]. Treg cells work to maintain immune homeostasis by suppressing excessive immune responses [24]. Recent research has revealed that Treg cells perform a significant role in inflammatory diseases of the CNS, such as ischemic stroke [25]. However, little is known about the potential role of Treg in pyroptosis following SCI.

In the present study, we discovered that post-spinal cord injury pyroptosis occurred primarily in microglia, along with a significant increase in Treg cell infiltration in spinal cord tissue. Our findings suggest that Treg cell deficiency causes widespread microglia pyroptosis and impaired motor function recovery following spinal cord injury. Mechanistically, Treg cells inhibit microglia pyroptosis by secreting exosomes, thereby promoting recovery of motor function after spinal cord injury. Finally, over-expression of miR-709 in Treg-exosomes significantly reduced the inflammatory response and improved motor recovery after spinal cord injury. These findings could help pave the way for the treatment of spinal cord injury and other neuroinflammation-related neurological disorders.

## Result

### Pyroptosis occures in microglia and Treg cell is increased after spinal cord injury

**Fig. 1:**
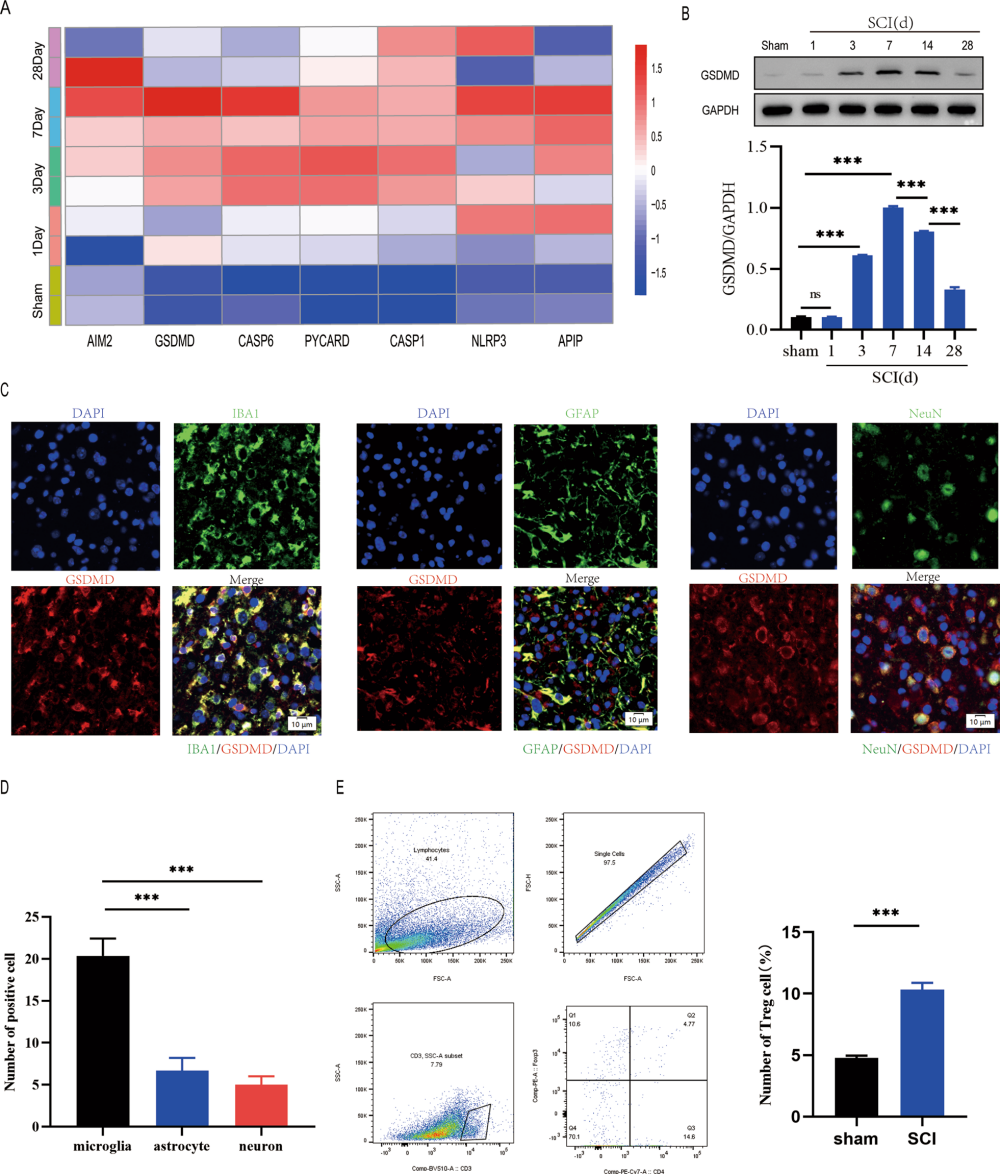
Pyroptosis occurs in microglia following spinal cord injury. **A** pyroptosis-related gene expression patterns at days 1, 3, 7, and 28 after SCI; **B** Representative immunoblots showing the level of GSDMD protein at Days 1, 3, 7, 14, and 28 following SCI. measurement of the relative GSDMD protein levels (n = 3); **C** Immunofluorescence of the spinal cord 7 days after injury reveals that GSDMD is mostly expressed in microglia (IBA1) but less in neurons (NeuN) or astrocytes (GFAP); **D** Quantification of fluorescence intensity of GSDMD at Day 7 postinjury(n = 3); **E** Flow cytometry demonstrating that Treg cell infiltration is significantly increased in the spinal cord 7 days after injury(n = 3)

To investigate the spatiotemporal characteristics of pyroptosis after spinal cord injury, we performed a serious of experiments. Bioinformatics analysis of GSE5296 revealed the expression of pyroptosis-related genes increased in mice with spinal cord injury than in sham-operated mice on day 1 and was highest on day 7, suggesting that pyroptosis peaked on day 7 after SCI(Fig. 1A). GSDMD protein levels were also markly increased on day 7(Fig. 1B). Since the spinal cord contains a variety of cell types, including astrocytes (GFAP^+^), microglia (IBA1^+^), and neurons (NeuN^+^), we investigated whether pyroptosis occurs in one or more kinds of cells after spinal cord injury. Immunofluorescence analysis revealed that pyroptosis mainly occured in microglia in the injured spinal cord and was lessly occured in other cell types(Fig. 1C, D). Furthermore,We ascertained a increas population of the Foxp3 + Treg cell in the injured spinal cord using flow cytometry at days 7 after spinal cord injury(Fig. 1E). These results suggest that Treg cell may be important in the regulation of microglia pyroptosis following spinal cord injury.

### Specific Treg-cell ablation in Foxp3^DTR^ mice promotes microglia pyroptosis in vivo

**Fig. 2:**
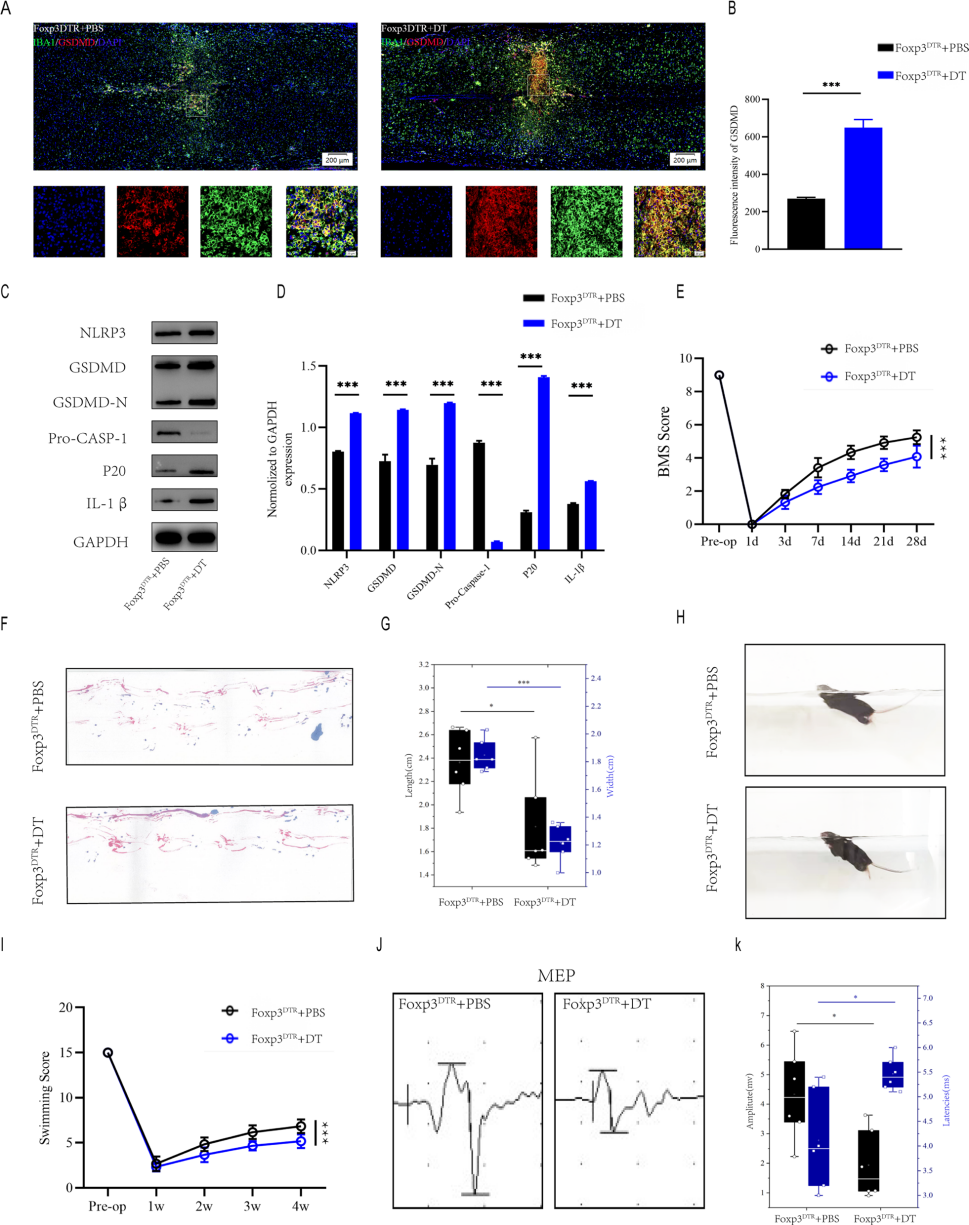
Knokout of Treg cell leads to widespread microglia pyroptosis and impairs functional recovery after SCI. **A** Representative immunofluorescence images for GSDMD(pyroptosis) and IBA1(microglia) expression in Foxp3DTR + PBS and Foxp3DTR + DT mice at Day 7 postinjury; **B** Quantification of fluorescence intensity at Day 7 postinjury(n = 3); **C** Representative immunoblots exhibiting levels of pyroptosis-related protein in injured spinal cord at days 7 postinjury in Foxp3DTR + PBS and Foxp3DTR + DT mice; **D** Quantification of relative levels of pyroptosis-related protein (n = 3); **E** BMS scores during 28 days of recovery after SCI demonstrate impaired functional recovery in Foxp3DTR + DT mice (n = 6); **F** Footprint analysis at Day 28 postinjury demonstrate impaired functional recovery in foxp3DTR + DT mice; **G** The footprints quantifcation of mice walking 28 days after SCI(n = 6); **H** Swimming test at Day 28 postinjury demonstrate impaired functional recovery in foxp3DTR + DT mice; **I** Louisville Swim Scale in Foxp3DTR + PBS and Foxp3DTR + DT mice at Day 28 postinjury(n = 6); **J** MEP analysis was used as electrophysiological assessment in Foxp3DTR + PBS and Foxp3DTR + DT mice at Day 28 postinjury; **K** Quantification of peak-to-peak MEP amplitudes and latencies in Foxp3DTR + PBS and Foxp3DTR + DT mice (n = 6)

To assess the effect of Treg cells on microglial pyroptosis after spinal cord injury, we selectively depleted Treg cells by diphtheria toxin (DT) injections in Foxp3^DTR^ transgenic mice that express the DT receptor under control of the Foxp3 promoter. Immunofluorescence of injury regions revealed that Foxp3^DTR^ + DT mice had more IBA1^+^/GSDMD^+^ microglia than Foxp3^DTR^ + PBS mice(Fig. 2A, B). Moreover, The severity of pyroptosis was also determined by using westernblot to detect NLRP3, GSDMD, GSDMD-N, Pro-CASP-1,caspase1(p20) and IL-1β levels in the injury center 7 days after spinal cord injury(Fig. 2C, D). Foxp3^DTR^ + DT mice have significantly more extensive microglia pyroptosis than Foxp3^DTR^ + PBS mice one week after SCI, implying that Treg cell knockout promote microglial pyroptosis in vivo. BMS score demonstrated that Foxp3^DTR^ + DT mice recovered significantly less than Foxp3^DTR^ + PBS mice during the 4-week post-SCI recovery process (Fig. 2E). Foxp3DTR + DT mice had less movementr coordination and less effective gait recovery, as shown by footprint assessment, supporting the findings of the BMS (Fig. 2F, G). Foxp3^DTR^ + DT mice also had poorer functional recovery in Swimming test (Fig. 2H, I).Furthermore, electrophysiological analysis revealed that Foxp3^DTR^ + DT mice had smaller amplitudes and longer latencies of motor evoked potentials (MEPs) after SCI than Foxp3^DTR^ + PBS mice(Fig. 2J, K). These behavioral tests indicate that deletion of Treg cell in mice following SCI results in poor functional recovery. These findings suggest that Treg cell deficiency causes extensive microglial pyroptosis and impairs functional recovery following spinal cord injury.

### Increased Treg-cell infiltration in spinal cord attenuates microglia pyroptosis in vivo

**Fig. 3:**
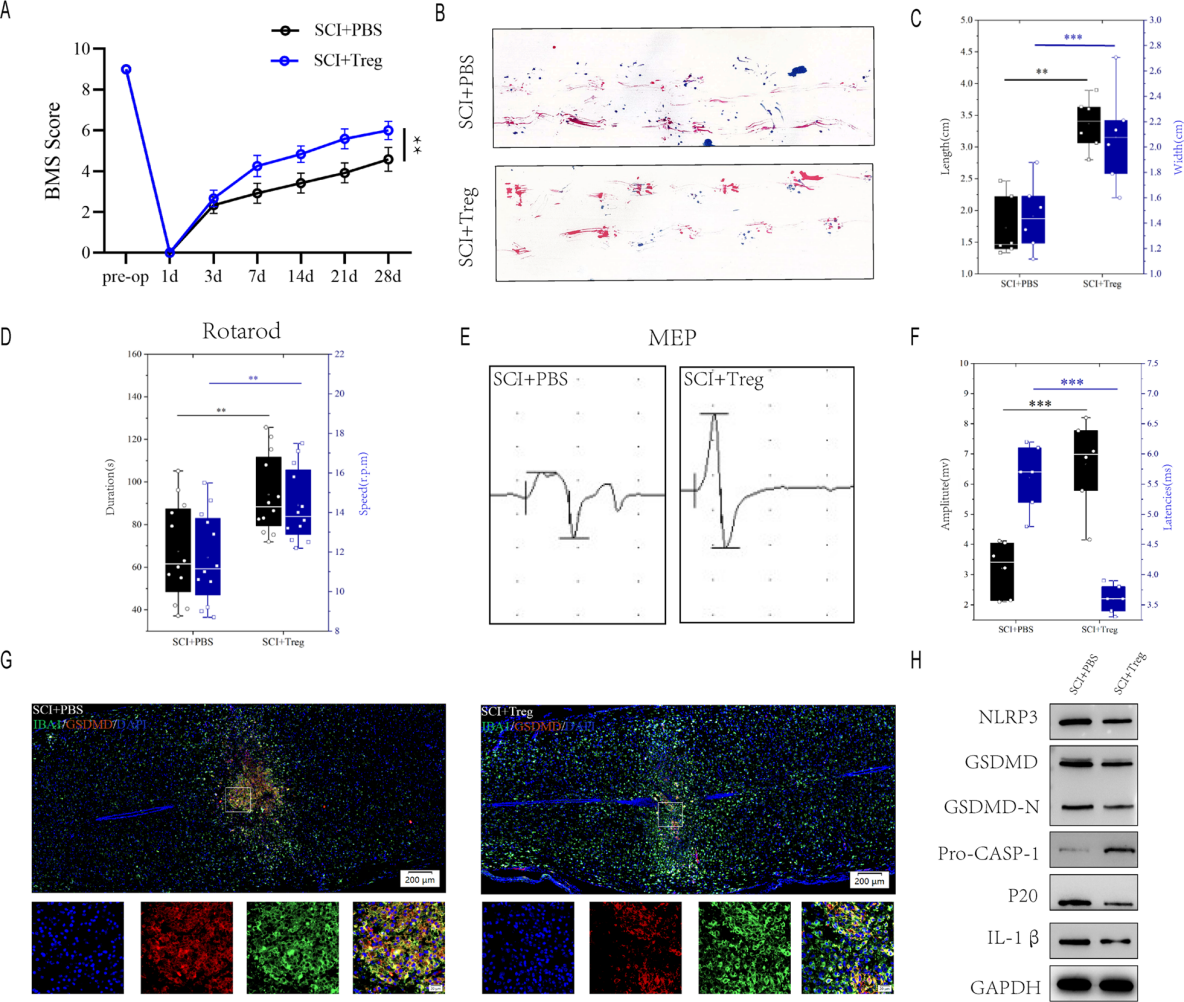
Increased-infiltration of Treg cell in spinal cord promotes functional recovery and limits microglia pyroptosis after SCI. **A** BMS scores during 28 days of recovery after SCI demonstrate better functional recovery in SCI + Treg mice (n = 6); **B** Footprint analysis at Day 28 postinjury demonstrate better functional recovery in SCI + Treg mice(n = 6); **C** The footprints quantifcation of mice walking 28 days after SCI; **D** Rotarod testsat Day 28 postinjury demonstrate better functional recovery in SCI + Treg mice(n = 12); **E** MEP analysis was used as electrophysiological assessment in SCI + PBS and SCI + Treg mice at Day 28 postinjury (n = 6); **F** Quantification of peak-to-peak MEP amplitudes and latencies in SCI + PBS and SCI + Treg mice. **G** Representative immunofluorescence images for GSDMD (pyroptosis) and IBA1 (microglia) expression in SCI + PBS and SCI + Treg mice at Day 7 postinjury. **H **Representative immunoblots showing levels of pyroptosis-related protein in injured spinal cord at days 7 postinjury in SCI + PBS and SCI + Treg mice

To further assess the effect of Treg cells on microglial pyroptosis after spinal cord injury, We injected Treg cells in the tail vein of mices immediately after spinal cord injury, and the infiltration of Foxp3 + cells in the spinal cord was significantly increased on day 7 after spinal cord injury (Figure S1A).BMS score demonstrated that SCI + PBS mice recovered significantly less than SCI + Treg mice during the 4-week post-SCI recovery process (Fig. 3A). SCI + PBS mice had less movementr coordination and less effective gait recovery, as shown by footprint assessment, supporting the findings of the BMS ((Fig. 3B, C). Rotarod testing for posterior limb and trunk equilibrium exhibited superior motor recovery in SCI + Treg mice (Fig. 3D). Electrophysiologic test also revealed that SCI + PBS mice had smaller amplitudes and longer latencies of motor evoked potentials (MEPs) after SCI than SCI + Treg mice (Fig. 3E, F). These behavioral tests indicate that increased infiltration of Treg cell in mice following SCI results in better functional recovery. SCI + Treg mice have significantly less microglia pyroptosis than SCI + PBS mice one week after SCI (Fig. 3G). A quantitative analysis of IBA1^+^/GSDMD^+^ regions at the injury site revealed that SCI + Treg mice had less IBA1^+^/GSDMD^+^ microglia than SCI + PBS mice (Additional file 1: Figure S1B). Moreover, The severity of pyroptosis was also determined by using westernblot to detect NLRP3, GSDMD, GSDMD-N, Pro-CASP-1,p20 and IL-1β levels in the injury center 7 days after spinal cord injury(Fig. 3H). SCI + Treg mice had significantly lower levels of NLRP3, GSDMD, GSDMD-N, caspase1(p20) and IL-1β expression than SCI + PBS mice (Additional file 1: Figure S1C). These findings suggest that increased Treg cell promotes functional recovery following spinal cord injury and attenuates microglial pyroptosis.

### Treg cell-derived exosomes attenuates pyroptosis in BV2 microglia in vitro

**Fig. 4:**
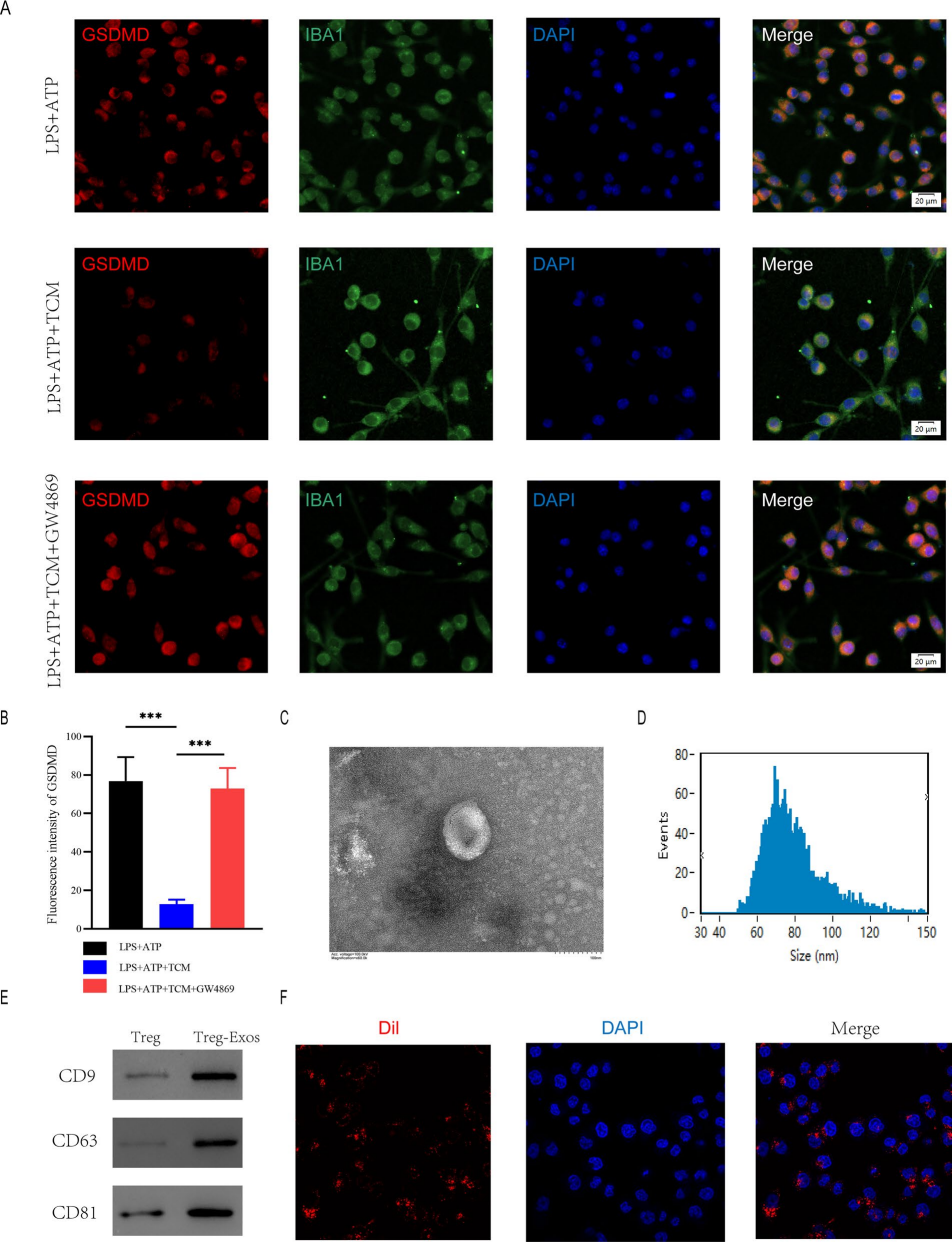
Treg-derived exosomes suppressed microglia pyroptosis. **A** Representative immunostaining image of IBA1 and GSDMD in the microglia; **B** Quantification of fluorescence intensity(n = 3); **C** Morphology of exosomes under TEM (50–150 nm); **D** NTA analysis exhibit exosomes size; **E** Western blot analysis of exosome surface markers; **F** The red fuorescence dye Dil-labeled exosomes was uptaken into microglia

To further investigate the mechanism of microglia pyroptosis inhibition by Treg cells, we collected Treg-cell conditioned medium(TCM) after Treg cells were stimulated with anti-CD3 (1ug/ml) and anti-CD28 (10ug/ml). BV2 cells were co-cultured with TCM before being treated with LPS + ATP. microglia were immunofluorescently stained for IBA1 and GSDMD to assess pyroptosis. GSDMD expression was considerably lower in the LPS + ATP + TCM group in comparison to the group that received only LPS + ATP treatment. Because exosomes contribute significantly in intercellular communication by transferring hereditary material, we speculated Treg cells attenuates pyroptosis in microglia by secreting exosomes. In order to prevent Treg cells from secreting exosomes, we used GW4869. Co-cultured TCM pre-treated GW4869 restored the expression levels of GSDMD (Fig. 4A, B).

### Exosomes derived from Treg cells aid in the recovering of motor performance and attenuates pyroptosis following SCI

To further explore the function of Treg cell-derived exosomes in the spinal cord injury micro-environment and the underlying effect on microglia pyroptosis, we extracted exosomes (Treg-Exos) from Treg cells culture supernatant and identified them using transmission electron microscopy (TEM), nanoparticle tracking analysis (NTA) and western blot. TEM showed classic nanoparticles with diameters between 50 and 150 nm, and NTA showed a semblable size distribution (Fig. 4C, D). Exosome surface markers like CD9, CD63, and CD81 were detected by Western blot (Fig. 4E). Exosomes that were Dil-labeled were taken up by microglia (Fig. 4F). The above investigations verified the exosome seperated from Treg supernatants.

**Fig. 5:**
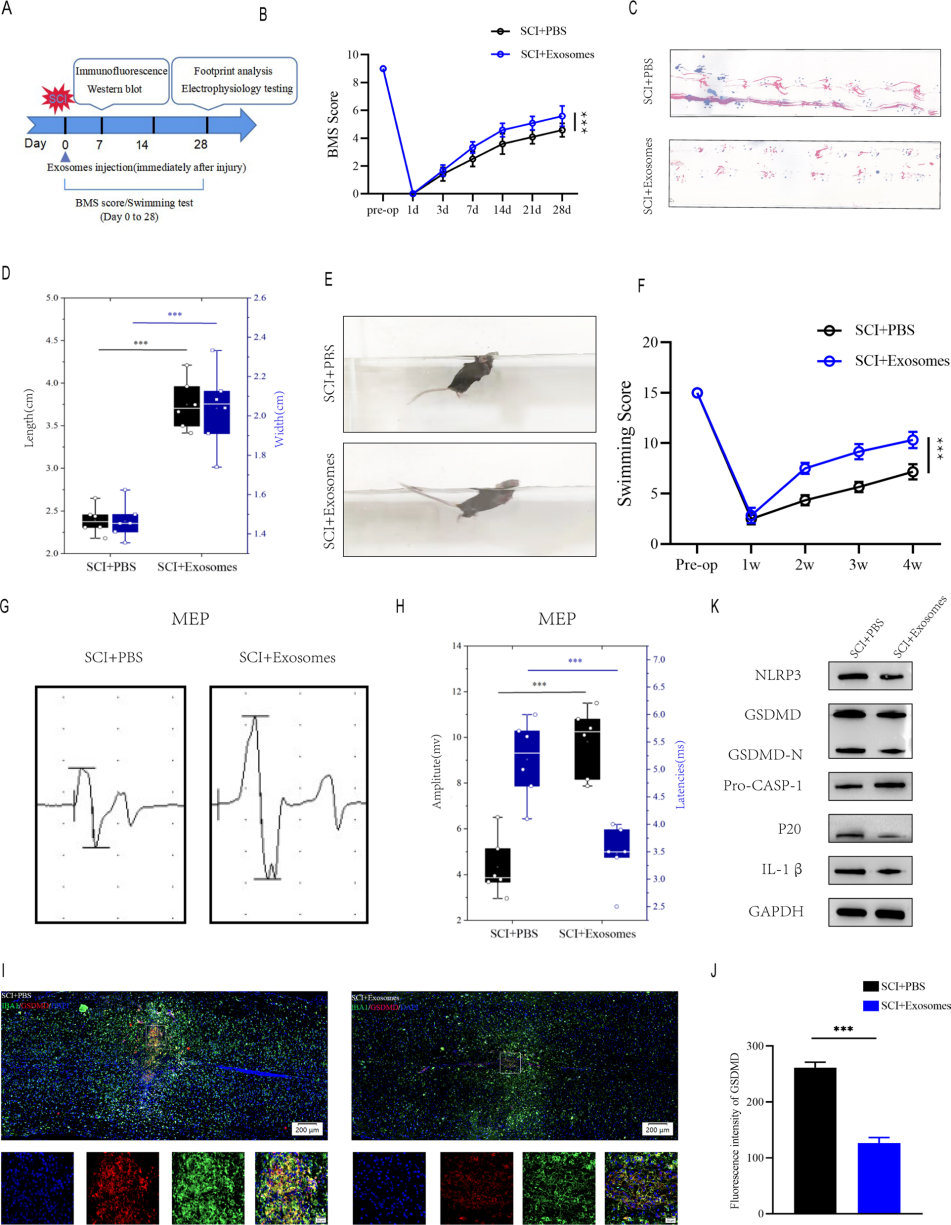
Exosomes derived from Treg cell promote motor function recovery and supress microglia pyroptosis after SCI in vivo. **A** The experimental scheme for exosomes injection after SCI; **B** BMS was used to functionally grade mice in different groups up to 28 days post-injury. **C** Footprint analysis at Day 28 postinjury demonstrate better functional recovery in SCI + Exosomes mice; **D** The footprints quantifcation of mice walking 28 days after SCI(n = 6); **E** Swimming test at Day 28 postinjury demonstrate better functional recovery in SCI + Exosomes mice; **F** Louisville Swim Scale score in SCI + PBS and SCI + Exosomes mice at Day 28 postinjury(n = 6); **G**MEP analysis was used as electrophysiological assessment in SCI + PBS and SCI + Exosomes mice at Day 28 postinjury; **H** Quantification of peak-to-peak MEP amplitudes and latencies in SCI + PBS and SCI + Exosomes mice (n = 6); **I** Representative immunofluorescence images for GSDMD(pyroptosis) and IBA1(microglia) expression in SCI + PBS and SCI + Exosomes mice at Day 7 postinjury; **J** Quantification of fluorescence intensity at Day 7 postinjury (n = 3); **K** Representative immunoblots showing levels of pyroptosis-related protein in injured spinal cord at days 7 postinjury in SCI + PBS and SCI + Exosomes mice

Exosomes were injected at once following SCI, and behavioral tests were conducted at the times specified (Fig. 5A). According to BMS behavioral analysis, Treg-Exos injection improved motor function scores in the hind limbs following spinal cord injury (Fig. 5B). Footprint analysis, swimming tests and MEPs all yielded similar results, with Treg-Exos injection resulting in longer strides, higher MEP amplitude, smaller body and water surface angles, and a more upturned tail(Fig. 5C–H). When Treg-exos-treated group were compared to PBS group, immunofluorescence staining revealed a decrease in IBA-1 + /GSDMD + cells and fluorescence intensity of GSDMD (Fig. 5I, J).Moreover, Treg-exos-treated group had significantly lower levels of NLRP3, GSDMD, GSDMD-N,p20 and IL-1β expression than PBS group(Fig. 5K and Additional file 1: Figure S2A).

Moreover, The results of immunofluorescence showed that Treg-exosomes had better therapeutic efficacy in terms of treating microglia pyroptosis compare with Treg cell treatment, which may be due to the characteristics of smaller particle size and higher membrane permeability of exosomes, which allowed them to easily cross the blood-spinal barrier, thus exerting a better effect on inhibiting microglia pyroptosis (Additional file 1: Figure S2B-C).

Taken together, these findings suggest that Treg-Exos can promotes functional recovery after SCI and decrease the onset of microglial pyroptosis.

### Exosomes deliver miR-709 to microglia

Treg-Exos promotes functional recovery after SCI and inhibits the onset of microglial pyroptosis, according to in vivo and in vitro studies. Exosomal miRNAs have been demonstrated in prior research to have regulating actions on target cells and may be crucial for the adjust of biological processes. miR-709 was screened by taking the intersection of the top 5 expressed miRNAs in Treg cells and Treg-Exos (Figure S3A). Moreover, miR-709 was markly increased in Treg-Exos-treated BV2 cells, indicating that exosomes can transfer miR-709 from Treg cells to BV2 cells (Additional file 1: Figure S3B).

### Exosomes suppressed microglia pyroptosis and motor function recovery after SCI by delivering miR-709

Because the above study demonstrated that Treg-derived exosomal miR-709 can be transmitted to microglia, we wondered if miR-709 could act as a biological messenger between Treg cells and microglia, regulating microglia pyroptosis and motor function recovery after SCI. To explore the function of exosomal miR-709 in the Treg-Exos-regulated microglia pyroptosis after SCI, miR-709 overexpression (miR-709^OE^) and knockdown (miR-709^KD^) Treg cells using a lentiviral-based method as well as the corresponding negative control (miR-NC^OE^ and miR-NC^KD^) were established.

**Fig. 6:**
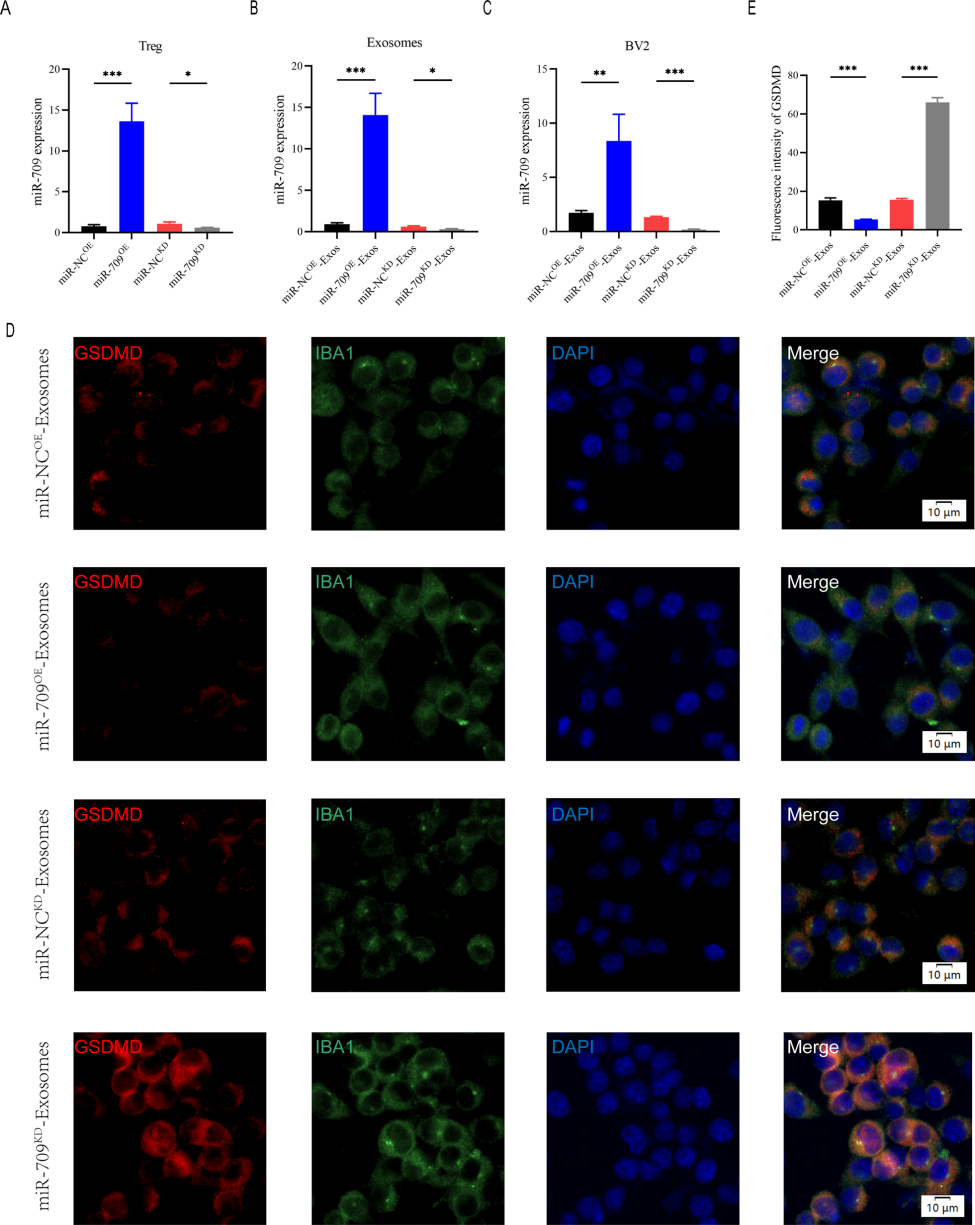
Treg-Exos supress pyroptosis in BV2 cells by delivering miR-709 in vitro. **A** Transfection efficiency of miR-709 overexpression and knockdown in Treg cells; **B** The relative expression of miR-709 in exosomes derived from Treg cells in indicated groups; **C** The relative expression of miR-709 in BV2 cells administered with miR-NC^OE^-Exos, miR-709^OE^-Exos, miR-NC^KD^-Exos and miR-709^KD^-Exos; **D**, **E** Representative immunostaining images and quantification of GSDMD fluorescence intensity in indicated groups

The transfection efficiency was confirmed using qRT-PCR (Fig. 6A). Exosomes were isolated from miR-NC^KD^-Tregs, miR-709^KD^-Tregs, miR-NC^OE^-Tregs, and miR-709^OE^-Tregs named miR-NC^KD^-Exos, miR-709^KD^-Exos, miR-NC^OE^-Exos, and miR-709^OE^-Exos, respectively. A significant decrease in the expression of miR-709 in miR-709^KD^-Exos compared with the miR-NC^KD^-Exos,while an evident increase in the expression of miR-709 in miR-709^OE^-Exos when compared with the miR-NC^OE^-Exos was observed (Fig. 6B). Furthermore, the miR-709 expression level in the target BV2 microglia in the miR-709^KD^-Exos treatment group showed a dramatic decrease in expression compared with the miR-NC^KD^-Exos treatment group. The 709 expression levels in the target BV2 microglia in the miR-709^OE^-Exos treatment group showed an increase in expression compared with the miR-NC^OE^-Exos treatment group (Fig. 6C). To assess pyroptosis, BV2 cells were then treated with LPS + ATP. Fluorescent intensity of GSDMD was markedly decreased by addition of miR-709^OE^-Exos compared with miR-NC^OE^-Exos treatment group, while miR-709^KD^-Exos treatment strongly enhanced GSDMD expression compared with miR-NC^KD^-Exos treatment group (Fig. 6D, E). Furthermore, expression of pyroptosis proteins detected by western blot demonstrated similar results with those discussed above (Additional file 1: Figure S4A-D).

**Fig. 7:**
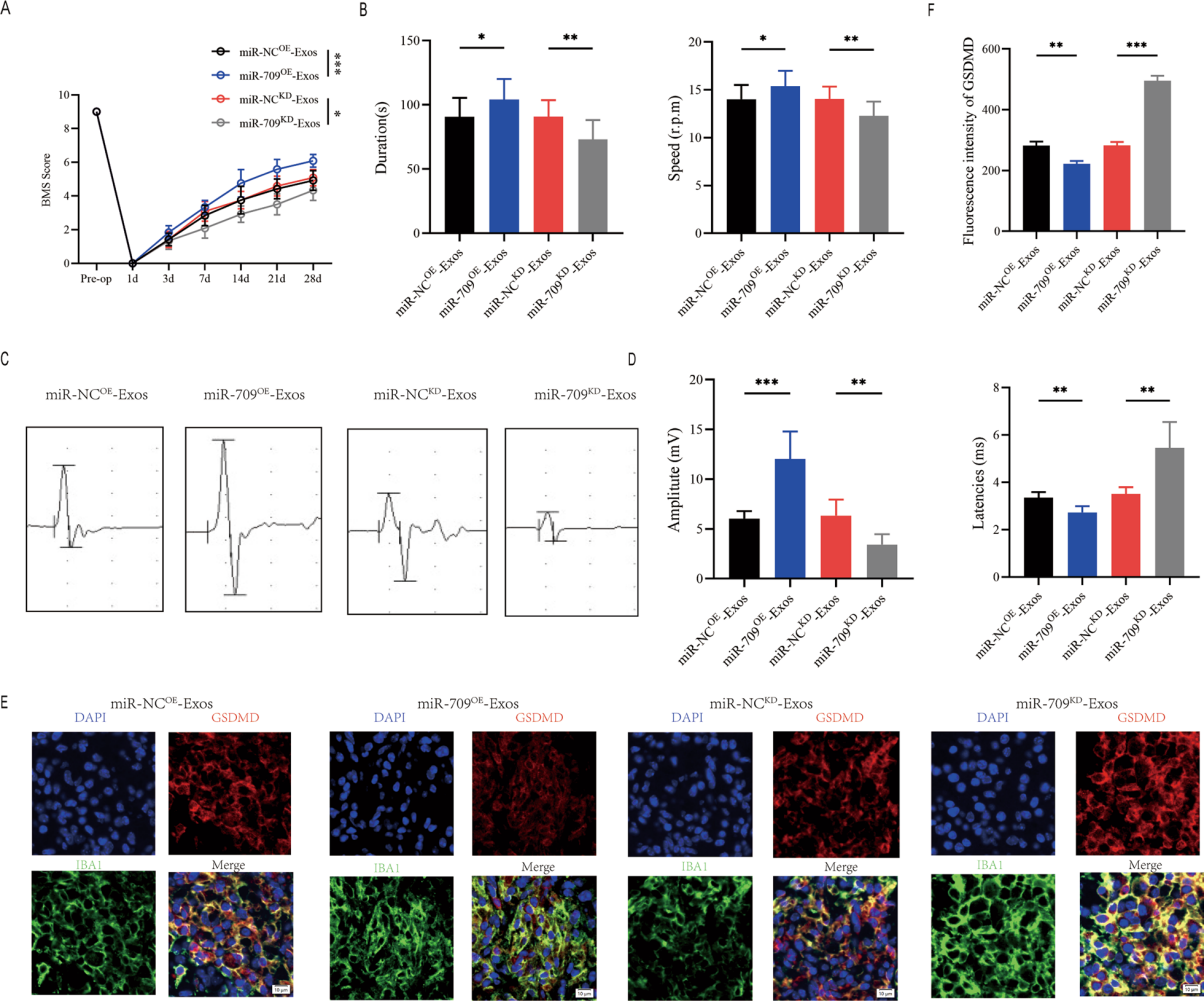
Treg-Exosomes promote motor function recovery and supress microglia pyroptosis by delivering miR-709 after SCI. **A** BMS was used to functionally grade mice in indicated groups up to 28 days post-injury; **B** Rotarod tests analysis of indicated groups at day 28 post-injury; **C** MEP analysis was used as electrophysiological assessment in indicated groups at Day 28 postinjury; **D** Quantification of peak-to-peak MEP amplitudes and latencies in indicated groups (n = 6); **E** Representative immunofluorescence images for GSDMD(pyroptosis) and IBA1(microglia) expression in indicated groups at Day 7 postinjury; **F** Quantification of fluorescence intensity at Day 7 postinjury (n = 3)

miR-709^OE^-Exosomes, miR-709^KD^-Exosomes and their negative control were respectively injected WT mice immediately after SCI, and behavioral assessments were conducted at the indicated times. According to BMS behavioral analysis, the miR-709^OE^-Exosomes enhanced the effect of Treg-Exos on improving hindlimb motor function following spinal cord injury, while miR-709^KD^-Exos treatment reduced the effect of Treg-Exosomes (Fig. 7A). Rotarod testing and MEPs all produced similar results (Fig. 7B–D). Immunofluorescence staining in the miR-709^OE^-Exos group compared to the miR-NC^OE^-Exos group revealed an decrease fluorescence intensity of GSDMD, while miR-709^KD^-Exos group compared miR-NC^KD^-Exos group exhibited an increased fluorescence intensity (Fig. 7E, F). These findings suggest that Treg-Exos, by delivering miR-709, inhibits microglia pyroptosis and promotes motor function recovery after spinal cord injury.

### miR-709 negatively regulates NKAP

**Fig. 8:**
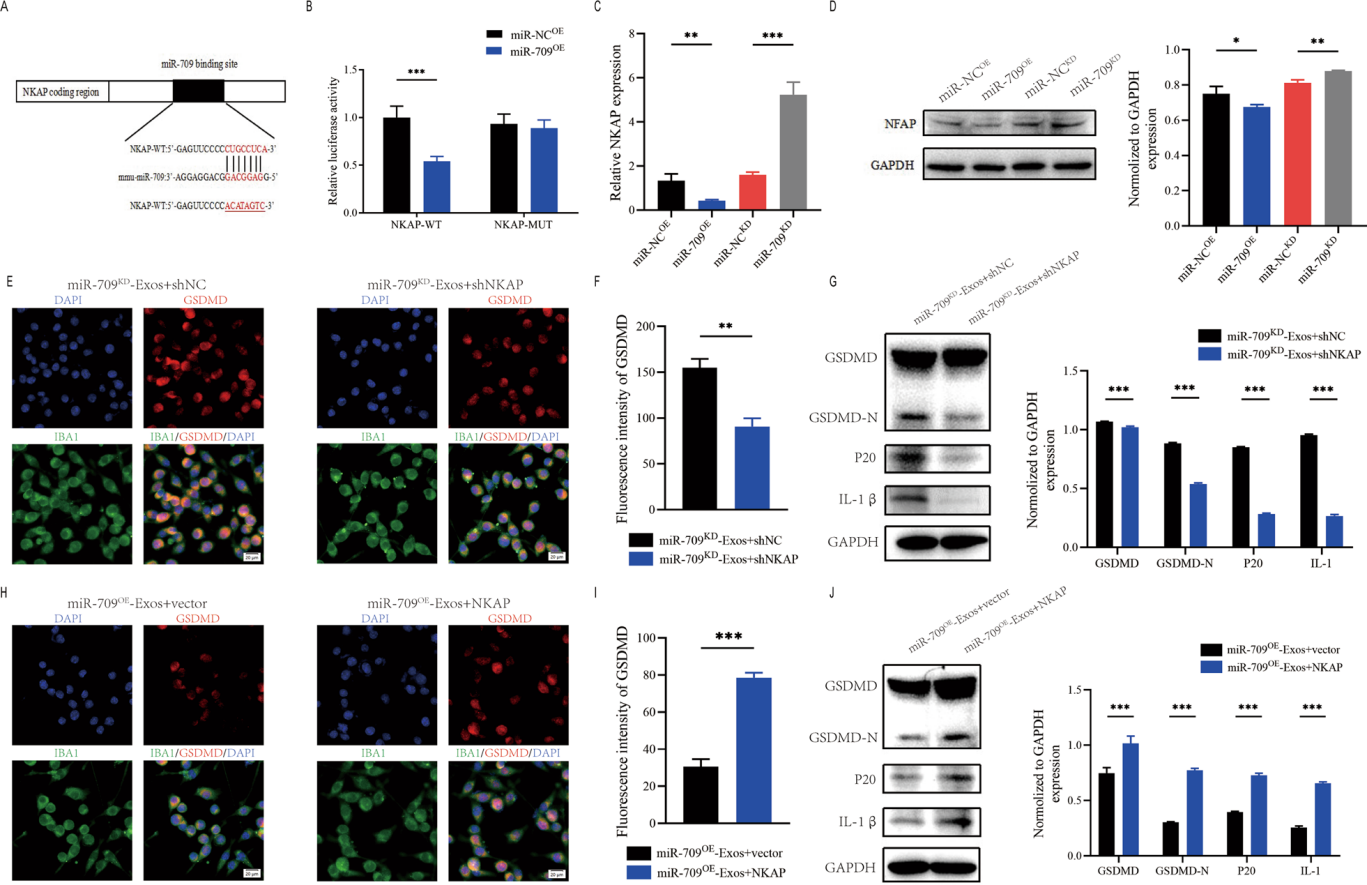
Exosomal miR-709 supress microglia pyroptosis by regulating NKAP expression. **A** Exosomal miR-709 regulates NKAP by directly targeting the 3′-UTR; **B** Luciferase report assay was performed to confirm NKAP is the target gene of miR-709; **C** The mRNA level of NKAP in BV2 cells after treatment with miR-709^OE^-Exos and miR-709^KD^-Exos; **D** The protein level of NKAP in BV2cells after treatment with miR-709^OE^-Exos and miR-709^KD^-Exos; **E**–**G **Rescue experiments for miR-709 inhibition were conducted by downregulating NKAP in microglia. microglia pyroptosis was detected by immunofuorescence (**E**, **F**) and western blot analysis (**G**); **H**–**J** Rescue experiments for miR-709 overexpression were carried out by the ectopic expression of NKAP in microglia. microglia pyroptosis was detected by immunofuorescence (**H**, **I**) and western blot analysis (**J**)

To investigate the underlying mechanism of action of exosomal miR-709. According to the online database of miRNA targets was used to search the predicted mRNA targets for miR-709. NKAP may be a potential target of miR-709 (Fig. 8A). Moreover, NKAP has been shown to play an active role in inflammation. To confirm that the NKAP 3'UTR is a direct target of miR-709, NKAP wild-type (WT) and mutant (MUT) 3'UTR sequences were created and cotransfected into 293T cells with miR-709 sequences.The luciferase reporter assay showed that miR-709 overexpression greatly reduced luciferase activity when co-transfected with WT-3' UTR of NKAP compared to control, but no inhibition activity of miR-709 was observed when co-transfected with MUT-3' UTR of NKAP (Fig. 8B). Further analysis using qRT-PCR and western blot assays showed that miR-709 knockdown increased NKAP mRNA and protein levels while miR-709 overexpression decreased NKAP expression(Fig. 8C, D).

### The impacts of miR-709^KD^-Exos on microglia are restored by NKAP silencing

To further explore the connection between exosomal miR- 709 and NKAP, some in vitro rescue trails were carried out.Using shRNA, the expression of NKAP was suppressed in BV2 cell. Results revealed that inhibiting NKAP during co-treatment with miR-709^KD^-Exos reduced the activation of microglia pyroptosis (Fig. 8E, F). Moreover, western blot supported these findings (Fig. 8G). As a result of these rescue experiments, it was demonstrated that shNKAP in microglia can abolish the facilitating role of miR709^KD^-Exos in promoting the microglia pyroptosis.

### The effects of miR-709^OE^-Exos on microglia are eliminated by overexpressing NKAP

NKAP was overexpressed by transfection with a NKAP lentivirus in microglia. Results demonstrated that overexpression of NKAP promoted the activation of microglia pyroptosis during co-treatment with miR-709^OE^-Exos(Fig. 8H, I). Western blot analysis also confrmed these results (Fig. 8J). Therefore, it was concluded that exosomal miR-709 suppresses the microglia  pyroptosis by targeting NKAP.

## Discussion

In the present study, we used loss-of-function and gain-of-function approaches to demonstrate the functional role of Treg cells in regulating microglia pyroptosis, and further investigated the specific ways in which Treg cells regulate microglia pyroptosis. Treg cells suppress microglia pyroptosis by secreting the exosome miR-709, which inhibits NKAP expression. Injection of Treg cells or Treg cell-derived exosomes inhibited microglia pyroptosis activation, resulting in improved functional recovery after spinal cord injury. Therefore, we believe Treg cells and their secreted exosomes are a promising therapeutic target for spinal cord injury.

Neuroinflammation following a spinal cord injury is a major cause of poor patient prognosis [26, 27]. Exploring the mechanisms that inhibit neuroinflammation after spinal cord injury is therefore critical for spinal cord injury treatment. Pyroptosis is a novel type of inflammatory cell death that has been identified as an important step in CNS neuroinflammation [28]. Treg cells appear to play an anti-inflammatory role in a variety of inflammatory diseases, including inflammatory bowel disease, amyotrophic lateral sclerosis, and arthritis [29–32]. The potential role of Treg cells in spinal cord injury, however, is unknown. According to bioinformatic analysis, pyroptosis was upregulated after spinal cord injury and peaked at day 7. To confirm the results of bioinformatics analysis, we examined the expression of GSDMD and discovered that it was significantly increased after spinal cord injury. According to single-cell sequencing analysis, pyroptosis after spinal cord injury occurs primarily in microglia. Immunofluorescence staining also showed that GSDMD was expressed almost exclusively in microglial cells. Furthermore, Treg cell infiltration was significantly increased after spinal cord injury, suggesting a role for Treg cells in microglial pyroptosis. Importantly, Treg cell transplantation significantly reduced microglia pyroptosis, resulting in improved functional recovery.

In vitro, we activated microglia with LPS + ATP to mimic the process of microglia pyroptosis and co-cultured them with TCM. Exosomes play a critical role in intercellular communication by transferring genetic material [33–36]. Treg cells have been shown to use the exosome pathway to maintain internal environment stability and to regulate intercellular interactions [37, 38]. Exosomes are captured by neighboring cells and used to regulate their biological processes [39]. Therefore, we hypothesized that exosome release is a critical mechanism by which Treg cells regulate microglia pyroptosis. The results show that Treg cell-derived exosomes can prevent microglia pyroptosis. In an in vivo study, by establishing a mouse spinal cord injury model, we demonstrated that exosome transplantation promotes behavioral functional recovery and inhibits microglia pyroptosis after spinal cord injury in mice. With the proven benefits of exosomes, we subsequently sought to determine the potential mechanisms by which exosomes promote functional recovery and inhibit microglia pyroptosis. Several studies have found that central nervous system exosomes perform biological functions on target cells by delivering specific miRNAs [40–42]. A bioinformatics analysis revealed that miR-709 was highly expressed in Treg cells and their exosomes. More importantly, after exosome treatment, miR-709 can be efficiently transferred to target microglia. According to a recent study, miR-709 plays an important role in the suppression of inflammatory responses [43]. However, no research has been published on the mechanism by which Treg cell-derived exosomes transport miR-709 and mediate inhibition of microglia pyroptosis after spinal cord injury. Through a series of in vitro and in vivo experiments, we found that downregulation of miR-709 in exosomes eliminated the beneficial effect of exosomes on SCI treatment, whereas overexpression of miR-709 in exosomes showed an increased beneficial effect. In conclusion, we suggest that miR-709-rich exosomes can inhibit microglia pyroptosis and promote behavior functional recovery after spinal cord injury, and that exosomes can act as biological carriers to deliver biologically functional miR-709 to recipient microglia.

To better understand the potential mechanisms of exosomal miR-709, we used bioinformatics tools to identify potential target genes of miR-709. Therefore, NKAP was selected for further study. NKAP has been shown to activate the NF-kB signaling pathway in a dose-dependent manner, and the NF-kB signaling pathway has been shown to play a key role in the activation of cellular pyroptosis [8, 44, 45]. The target gene was validated with a luciferase reporter gene. Western blot analysis revealed that NKAP protein levels was down-regulated upon overexpression of miR-709 and up-regulated upon knockdown of miR-709 in microglia. To further confirm that NKAP is a target gene of miR-709, we conducted a series of gain-of-function and loss-of-function experiments. The results showed that knockdown of NKAP in microglia reversed the detrimental effects of suppressing miR-709 expression in exosomes, while overexpression of NKAP counteracted the observed beneficial effects.

Although our results suggest that the Treg cell-derived exosome miR-709 plays a key role in the inhibition of microglia pyroptosis, other genes that may play a therapeutic role alone or in combination with exosomes cannot ruled out. The exact mechanism by which Treg cell-derived exosomes promote recovery of behavioral function after spinal cord injury in mice will be further explored in future studies.

Taken together, this study shows that Treg cells and their sourced exosomes can inhibit microglia pyroptosis and thus promote the recovery of motor function after spinal cord injury. These nano-sized exosomes can effectively deliver therapeutic exosomal miRNAs to the damaged spinal cord through the blood-spinal cord barrier, and thus have good potential for clinical application. Thus Treg cell, Treg cell-derived exosomes and miRNAs may provide a new therapeutic tool for the treatment of spinal cord injury.

## Material and methods

### Animals

Foxp3DTR mice acquired fromShanghai Nanfang Model Biotechnology Co., LTD (Shanghai, China). For creating Treg cell knockout mice, Foxp3DTR mice were injected with diphtheria toxin(DT). The First Affiliated Hospital of Nanjing Medical University's Animal Committee gave its approval to all of the experiments.

### SCI model

Mice between 8 and 10 weeks old were used to create a SCI animal model. In brief, isoflurane inhalation was used to sedate the mice before laminectomy was performed to disclose the spinal cord at T8. In brief, the spinal cord was then struck by a rod that weighed 5 g that was dropped from an altitude of 6.5 cm using an impactor to cause SCI. The muscles were sutured and the skin was stitched shut right away after injury. Up until reflexive control of bladder function was reestablished, we manually emptied the mice's bladders three times per day.

### Behavioral tests

Mice were kept in 12-h light–dark cycles with unlimited access to food and water. Prior to behavioral testing, all mice were given a 1-h acclimatization period in the testing space or equipment. The use of blind scoring ensured that observers were unaware of groups.

Neural function was assessed at 1, 3, 7, 14, and 28 days after SCI using the BMS score for mobility. The scale went from 0 (completely paraplyzed) to 9 (normal).

To assess coordination and balance of injured mice, we used a rotating object that could be speeded up from zero to fourty revolutions per minute (rpm). Each mouse was given access to one practice trial, once test trials, and a half hour break between each the two experiment.

Analyses of footprints were performed as previously mentioned [46]. The mouse's anterior and posterior limbs were dyed blue and red, respectively, and only when they ran at a steady velocity were stride lengths and widths measured.

The injured mouce undergone a swimming trial to gauge their return to motor function. Mice were taught to swim across the glass tank from one end to the other. The motor function were all evaluated using the Louisville Swim Scale.

Electromyography was used to evaluate MEPs of the mice at Day 28 postinjury. A stimulation electrode was placed at the rostral ends of the spinal cord exposed by surgery, the recording electrode was placed at the flexor of the biceps femoris, the reference electrode was inserted at the distal tendon of the hind limb muscle, and the ground electrode was placed under the skin. A single square wave stimulus (10 mA, 0.5 ms, 1 Hz) was used.

### Immunofluorescence staining assays

The injured spinal cords were then taken out and fixed overnight in 4 percent paraformaldehyde after the mice hearts had been perfused with 0.9 percent saline and then 4 percent paraformaldehyde. The samples were cut into 10 μm thick slices after being dehydrated. Slices were blocked with 10% BSA before being cultured with the primary antibodies anti-IBA1 and anti-GSDMD for an overnight period at 4 °C, followed by an hour at room temperature with the secondary antibodies.

Cells were fixed in 4 percent paraformaldehyde for 30 min, permeabilized with 0.05 percent Triton X-100, and then blocked with 5 percent BSA to perform cell immunofluorescence staining. The cells were then cultured with primary antibodies (anti-IBA1 and anti-GSDMD) overnight at 4 °C before being cultured with secondary antibodies. Nuclei were counterstained with DAPI following three PBS washes.

### Microarray data

GSE5296 provides transcriptional data for specific time points after spinal cord injury in mice. miRNA sequencing data in Treg cells and Treg-Exos were obtained from GSE60615.

### Cell culture

The murine BV2 microglial cell line was obtained from Shanghai Cell Research Center (Shanghai, China). The cells were cultured in DMEM (4.5 g/L glucose) containing 10% FBS and 1% penicillin/streptomycin at 37 °C in a 5% CO2 atmosphere. When the cells reach to approximately 80% confluence, they were digested with trypsin and passaged for additional experiments. LPS and ATP were used to induce BV2 cell pyroptosis.

### Cell depletion

Diphtheria toxin (DT, ip, 0.05 μg/g body weight) was injected 3 days prior to spinal cord injury to deplete Treg cells, and repeated every 3 days to maintain Treg cell depletion until 28 days after spinal cord injury.

### Treg cell isolation and adoptive transfer

Spleen, inguinal and axillary lymph nodes were harvested from uninjured mice (8–10-week-old) and pooled together to prepare single cell suspensions. CD4 + CD25 + Treg cells were isolated using a mouse Treg cell isolation kit (Miltenyi Biotec) according to the manufacturer’s instructions. The isolation was performed in a two-step procedure with a negative selection on CD4 + cells and a positive selection on CD25 + cells. For in vivo studies, 3 × 10^6^ freshly isolated Treg cells were transferred intravenously to recipient mice after spinal cord injury through the tail vein. Control mice received an equivalent volume of phosphate-buffered saline (PBS).

### Flow cytometry

Animals were euthanized and perfused with cold saline. Spinal cord homogenates were prepared with the Neural Tissue Dissociation Kit (T) using a gentle MACS dissociator with heaters (Miltenyi Biotec) following the manufacturer’s instructions. The suspension was passed through a 70-μm cell strainer (Thermo Fisher Scientific), and resuspended in 30% Percoll. Single cell suspensions were separated from myelin and debris by centrifugation (500 g, 30 min, 18 °C) on a 30–70% Percoll gradient. Cells at the interface were collected and washed with Hank’s balanced salt solution (HBSS; Sigma–Aldrich) containing 1% fetal bovine serum (Sigma–Aldrich) and 2 mM EDTA (Sigma–Aldrich). Single cell samples were first incubated with antibodies to surface antigens for 30 min on ice at 4 °C in the dark. After two washes, cells were fixed and permeabilized with Fixation/Permeabilization Diluent & Concentrate according to the manufacturer’s protocol. Fluorochrome compensation was performed with single-stained UltraComp eBeads. Flow cytometry was performed by MACSQuant Analyzer 10 (Miltenyi Biotec, Germany) and data were analyzed with FlowJo software.

### Exosomes isolation, identification and injection

Exosomes were taken out of the culture supernatant of Treg cells that had previously been cultured to exosome-depleted media. The supernatant was then gathered and centrifuged at 300 g and 2000 g for 10 min. To get rid of cellular debris, the cell supernatant was filtered through a 0.22 m filter after centrifugation. The supernatant was centrifuged at 4000 g until the upper compartment's volume reached 200 μl. The liquid in the upper chamber was loaded onto a 30 percent sucrose/D2O cushion and subjected to ultracentrifugation at 100,000 g for 60 min at 4 C in order to purify exosomes. The morphology of exosomes was examined using a transmission electron microscope (TEM). The diameter and number of exosomes were measured using nanoparticle tracking analysis (NTA).Exosome surface biomarkers were examined using a western blot. Mice received intrathecal injection of Treg-derived exosomes (100 µg in 5 µL) after spinal cord injury through.Control mice received an equivalent volume of phosphate-buffered saline (PBS).

### Exosome uptake by microglia

We used fluorescent exosome labeling in accordance with the manufacturer's recommendations. A 4-mg/mL Dil solution (Molecular Probes, OR, USA) was added to PBS containing exosomes and cultured. Excess dye were centrifuged at 100,000 g for one hour at 4 °C to remove it. The microglia were co-cultured with these Dil-labeled exosomes for 24 h, after which the cells were washed in PBS and fixed in 4 percent paraformaldehyde. The uptake of Dil-labeled exosomes by microglia was observed using laser confocal microscopy.

### Western blot analysis

RIPA lysis and extraction buffers were used to extract proteins from cells and spinal cord tissues. The BCA analysis was used to obtain protein concentration. SDS-PAGE was used to separate equal amounts of protein, which was then transmitted to PVDF films and cultured with primary antibodies overnight at 4 °C after being blocked with 5% BSA. After that, membranes were cultured with the secondary antibody for 2 h at room temperature. Using the ECL reagent from Thermo Fisher Scientific, immunolabeled bands were seen, and the expression of target protein bands was semi-quantified.antibodies as follows: anti-NLRP3 (1:1000), anti-Caspase-1 (1:1000), anti-Cleaved-Caspase-1 (1:1000), anti-GSDMD (1:1000), anti-GSDMD-N (1:1000), anti-IL-1β (1:1000), anti-NKAP(1:1000), and anti-GAPDH (1:2000).

### Luciferase reporter assay

Hippo Biotechnology Co., LTD. created sequences that correspond to the 3′-UTR of NKAP mRNA and contain either wild-type (WT) or mutated (MUT) miR-709 binding sequences (Nanjing, China). To create the NKAP3′-UTR reporter constructs, these sequences were cloned into the FseI and XbaI restriction sites of the pGL3 luciferase control reporter vector (Promega, Madison, WI, USA) (pGL3-WT-NKAP and pGL3-MUT-NKAP). Before transfection, HEK293T cells (ATCC, USA) were seeded in 24-well plates and given a 24-h incubation period. The miR-709^OE^ or negative control-transfected HEK293T cells were seeded into 96-well plates along with 100 ng of either the pGL3-WT-NKAP or pGL3-MUT-NKAP 3′-UTR. Using the Dual Luciferase Reporter Assay System, luciferase activity was assessed 48 h after transfection and normalized to firefly luciferase activity.

### RNA extraction and qRT-PCR

TRIzol reagent was used to extract the total RNA from cells and exosomes (Invitrogen, Carlsbad, CA, USA). Hairpin-itTM miRNA qPCR Quantitation Kit (GenePharma, China) and PrimeScript RT reagent Kit (Takara, Japan) were used to create the cDNA for miRNA and mRNA, respectively. The TB Green® Premix Ex TaqTM kit was then used to carry out the qRT-PCR assay (Takara, Japan). The expression levels of mRNA and miRNA were compared to GAPDH and U6, respectively. The 2 − ΔΔCT method was used to compute the relative expression.

### Statistical analyses

At least 3 repeated experiment were used to generate the data, which are presented as mean ± standard deviation. Statistical analysis was performed using GraphPad software 8.0. When comparing two groups, the unpaired, two-tailed Student's t-test was used. When comparing groups larger than two, one or two-way ANOVA was used. A statistically significant result was one with a p value < 0.05. (*p < 0.05;**p < 0.01;***p < 0.001).

## Supplementary Information

**Additional file 1: Figure S1.** (A) Representative flow cytometry in SCI + PBS group and SCI + Treg group; (B) Quantification of fluorescence intensity of GSDMD in SCI + PBS group and SCI + Treg group; (C) Quantification of western blot of pyroptosis-releative protein in SCI + PBS group and SCI + Treg group.

**Additional file 2: Figure S2.** (A) Quantification of western blot of pyroptosis-releative protein in SCI + PBS group and SCI + Exosomes group; (B) Representative immunofluorescence images for GSDMD expression in SCI + Treg group and SCI+Exosomes group; (C) Quantification of fluorescence intensity of GSDMD in SCI + Treg group and SCI+Exosomes group.

**Additional file 3: Figure S3.** (A) The intersection of the top 5 expressed miRNAs in Treg cells and Treg-Exos; (B) miR-709 was markly increased in Treg-Exos-treated BV2 cells.

**Additional file 4: Figure S4.** (A) Representative western blot of pyroptosis-releative protein in different exosomestreated group; (B–D) Quantification of western blot of pyroptosis-releative protein in different exosomes-treated group.

## Data Availability

The datasets analyzed during the current study are publicly available and can be obtained from the corresponding author upon reasonable request.

## Abbreviations

SCI: Spinal cord injury
CNS: Central nervous system
PRRs: Pattern recognition receptors
PAMPs: Pathogen-associated molecular patterns
DAMPs: Damage-associated molecular patterns
GSDMD: Gasdermin D
Foxp3: Forkhead box p3
DT: Diphtheria toxin
DTR: Diphtheria toxin receptor
LPS: Lipopolysaccharides
ATP: Adenosine triphosphate
GAPDH: Glyceraldehyde-3-phosphate dehydrogenase
