# Supplementary figures and images for: Treg cell-derived exosomes miR-709 attenuates microglia pyroptosis and promotes motor function recovery after spinal cord injury
Source: J Nanobiotechnology. 2022 Dec 13;20:529. doi: 10.1186/s12951-022-01724-y (PMC9745961; doi:10.1186/s12951-022-01724-y)

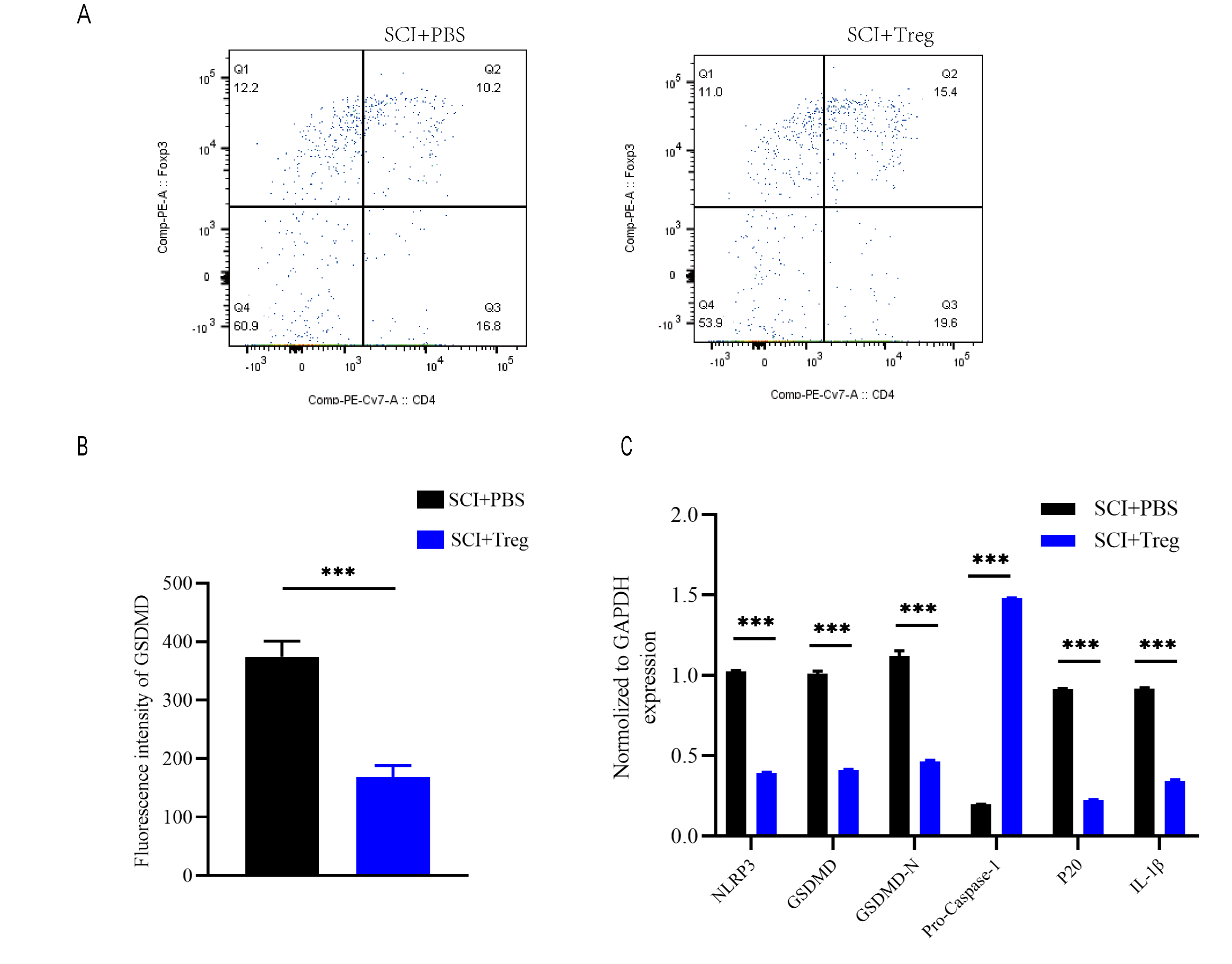

Supplement: Supplementary file 1 — Additional file 1: Figure S1. (A) Representative flow cytometry in SCI + PBS group and SCI + Treg group; (B) Quantification of fluorescence intensity of GSDMD in SCI + PBS group and SCI + Treg group; (C) Quantification of western blot of pyroptosis-releative protein in SCI + PBS group and SCI + Treg group. [file 12951_2022_1724_MOESM1_ESM.png]

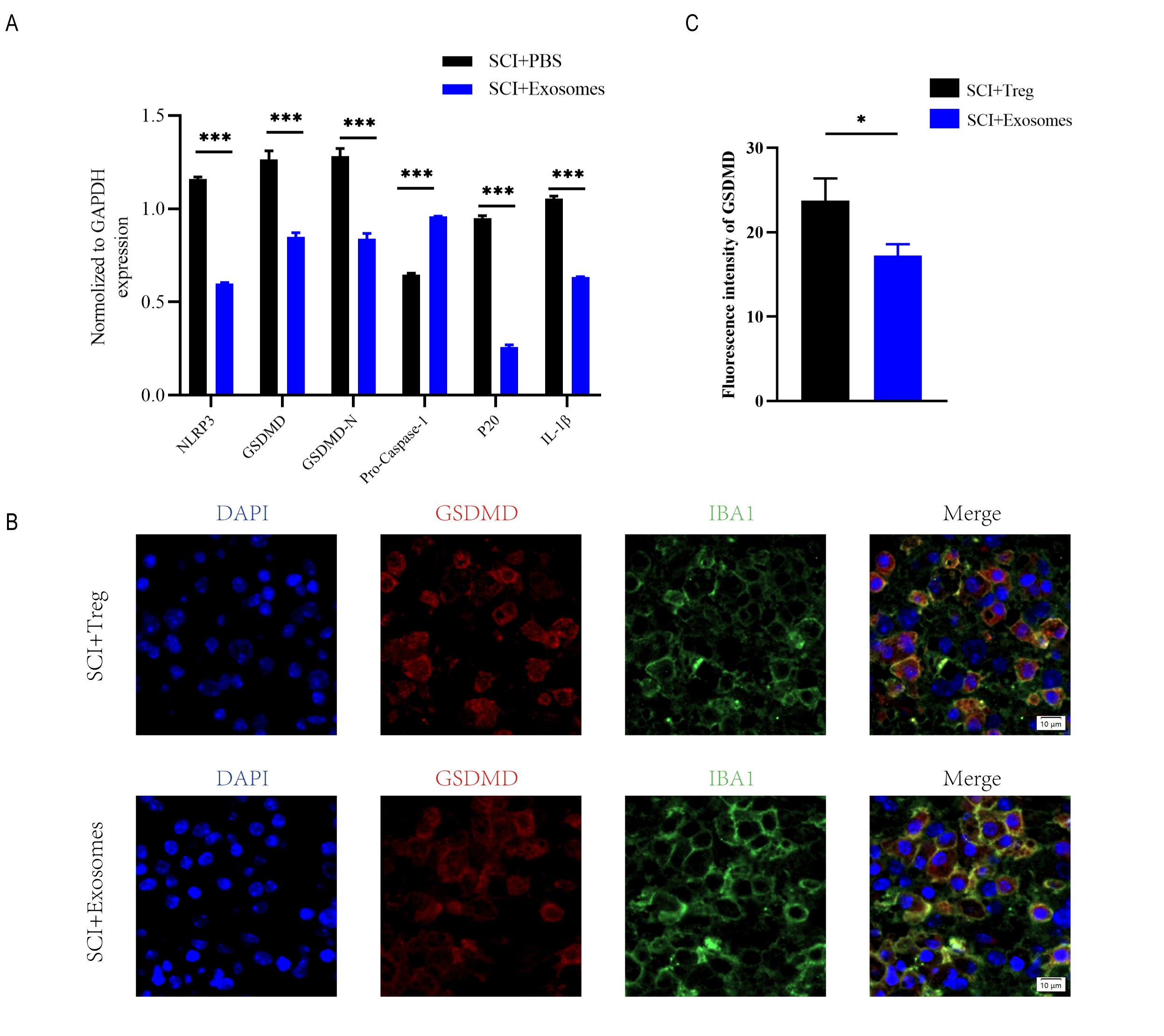

Supplement: Supplementary file 2 — Additional file 2: Figure S2. (A) Quantification of western blot of pyroptosis-releative protein in SCI + PBS group and SCI + Exosomes group; (B) Representative immunofluorescence images for GSDMD expression in SCI + Treg group and SCI+Exosomes group; (C) Quantification of fluorescence intensity of GSDMD in SCI + Treg group and SCI+Exosomes group. [file 12951_2022_1724_MOESM2_ESM.png]

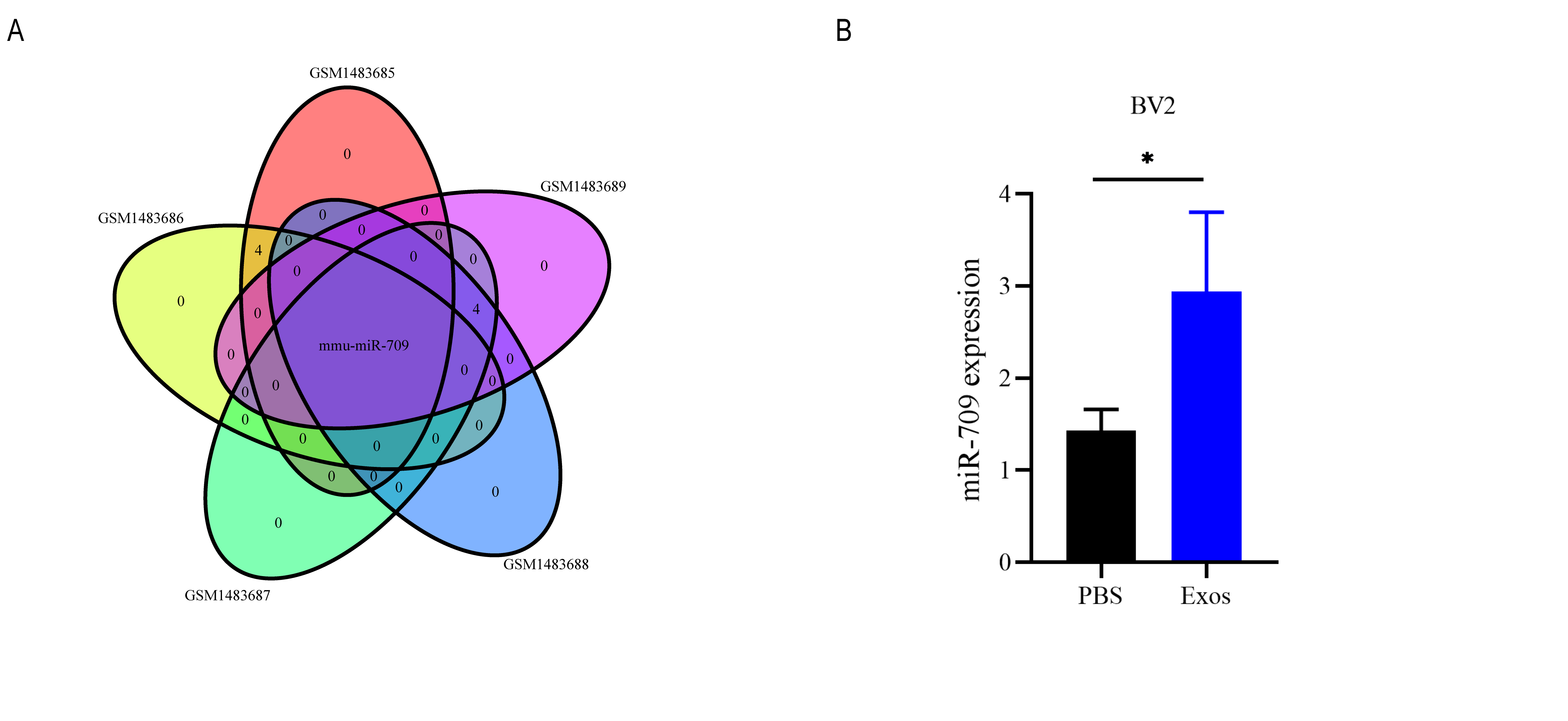

Supplement: Supplementary file 3 — Additional file 3: Figure S3. (A) The intersection of the top 5 expressed miRNAs in Treg cells and Treg-Exos; (B) miR-709 was markly increased in Treg-Exos-treated BV2 cells. [file 12951_2022_1724_MOESM3_ESM.png]

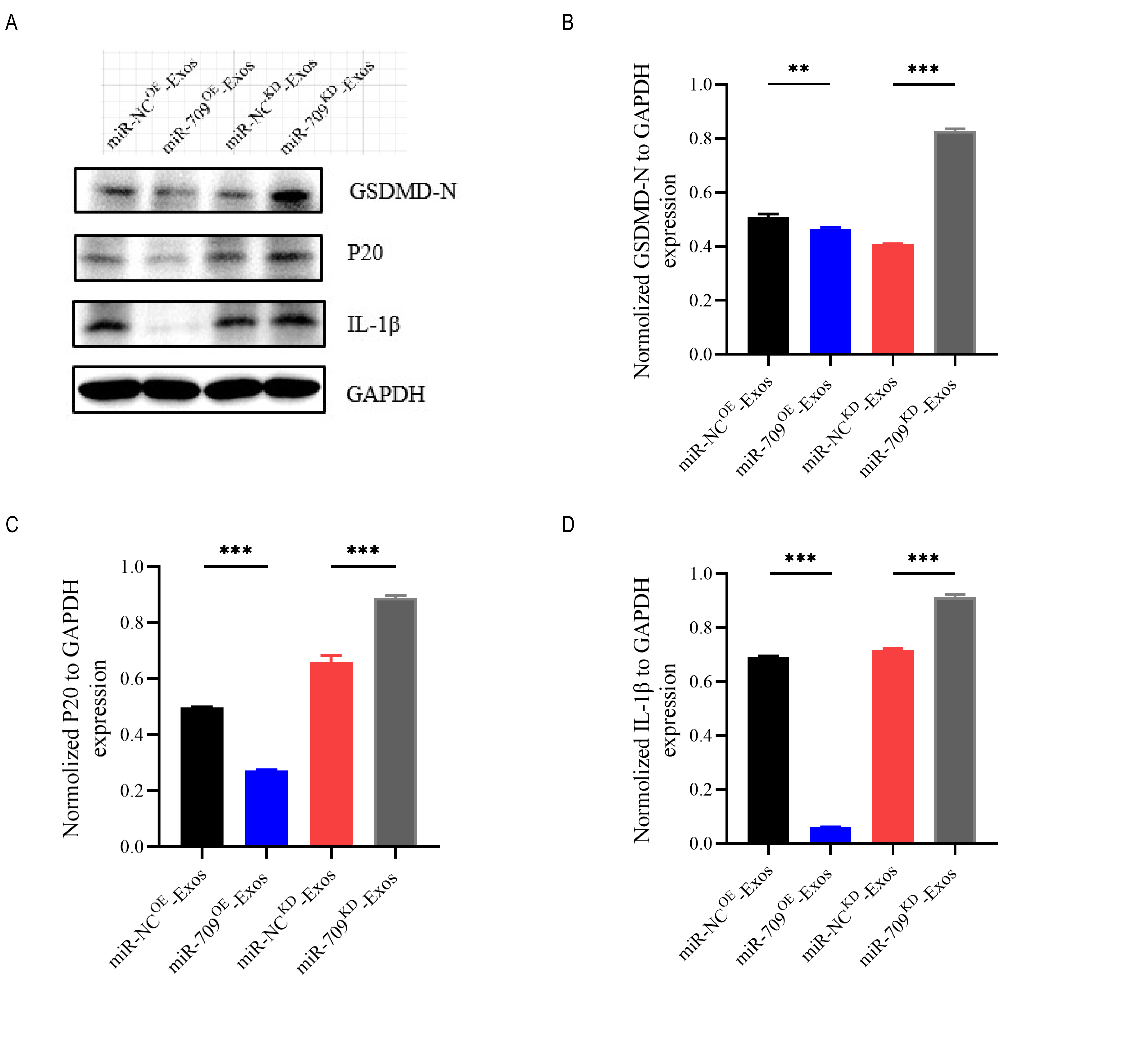

Supplement: Supplementary file 4 — Additional file 4: Figure S4. (A) Representative western blot of pyroptosis-releative protein in different exosomestreated group; (B–D) Quantification of western blot of pyroptosis-releative protein in different exosomes-treated group. [file 12951_2022_1724_MOESM4_ESM.png]
